# Mineral Accumulation and Physiological Responses of Two Halophytes, *Caroxylon vermiculatum* and *Mesembryanthemum nodiflorum*: Implications for Phytoremediation in Contaminated Coastal Environments

**DOI:** 10.3390/plants15182743

**Published:** 2026-09-08

**Authors:** Dhouha Belhadj Sghaier, Hasna Ellouzi, Houyem Abderrazak, Fourat Akrout, Mohsen Hanana, Monia EL Bour

**Affiliations:** 1 National Institute of Marine Sciences and Technologies, University of Carthage, Tunis 2025, Tunisia; fouratakrout@yahoo.fr (F.A.); moniaelbour@gmail.com (M.E.B.); 2 Laboratory of Extremophile Plants, Centre of Biotechnology of Borj Cedria, Hammam-Lif 2050, Tunisia; ellouzihasnaa@gmail.com (H.E.); mohsen.hnana@cbbc.rnrt.tn (M.H.); 3 National Institute of Research and Physicochemical Analysis, Sidi Thabet Technopole, Ariana 2020, Tunisia; houyem.snani81@gmail.com

**Keywords:** halophytes, salinity, heavy metals, phytoremediation, phytostabilization, antioxidant system, phenolic compounds

## Abstract

Halophytes are recognized for their adaptive capacity and potential applications in phytomanagement and ecosystem restoration. This study investigates the comparative physiology and antioxidant responses of two native halophytes, *Caroxylon vermiculatum* (L.) and *Mesembryanthemum nodiflorum* L., growing naturally in saline and metal-affected coastal environments. A comprehensive set of physiological and biochemical parameters was assessed, including macro- and microelements (Na, K, Ca, Fe, Zn, Cd), photosynthetic pigments (chlorophyll a, chlorophyll b, and carotenoids), soluble sugars, and proteins. In addition, oxidative stress markers (hydrogen peroxide, H_2_O_2_, and malondialdehyde, MDA), enzymatic antioxidants (superoxide dismutase, SOD, catalase, CAT, and guaiacol peroxidase, GPX), and non-enzymatic antioxidants (total phenolics, flavonoids, and proanthocyanidins) were evaluated. Antioxidant activities, including DPPH (2,2-diphenyl-1-picrylhydrazyl) radical scavenging and reducing power, were also measured. The results revealed clear species-specific adaptive strategies. *Mesembryanthemum nodiflorum* exhibited higher accumulation of Na and K, together with elevated levels of carotenoids and oxidative stress markers. This species showed translocation factors (TF > 1) for Na (~1.60) and K (~1.90), indicating efficient ion transport to aerial parts, while displaying low bioconcentration (BCF < 0.5 for most trace elements) and biological accumulation factors (BAF < 1), suggesting limited capacity for heavy metal accumulation. In contrast, *Caroxylon vermiculatum* showed higher concentrations of chlorophylls, carotenoids, phenolic compounds, flavonoids, and proteins, along with stronger superoxide dismutase activity. It also exhibited lower translocation of trace elements (TF < 1) and higher root retention of metals (BCF up to ~0.5), indicating a more effective exclusion and detoxification strategy. Overall, these findings demonstrate that *M. nodiflorum* relies on ion accumulation and translocation, whereas *C. vermiculatum* exhibits stronger ion regulation and antioxidant protection. Given the moderate BAF and BCF values observed, both species are more likely to contribute to phytomanagement through ion regulation and phytostabilization rather than efficient phytoextraction.

## 1. Introduction

Soil salinization, which represents one of the most severe constraints to agricultural productivity worldwide, is projected to intensify in the coming decades due to climate change and unsustainable land management practices [1,2]. Currently, more than 400 million hectares of agricultural land are significantly affected by salinity [3,4], with over 6% of the world’s land classified as salt-affected [5]. Alarmingly, approximately 250 million hectares of agricultural soils suffer from salt saturation [6]. The extent of salinity-affected soil continues to expand, increasing annually by nearly 10% of arable land, as reported by Kumar and Sharma [7]. In Europe alone, salt-affected soils cover approximately 24 million hectares, representing about 2.05% of the global salt-affected area (1171.8 Mha), a figure amplified by rising temperatures and increased drought intensity driven by global climate change [2,4,8].

In addition to salinity, the contamination of soil by heavy metals (HMs) represents another major environmental and agricultural challenge. Despite HMs coming from rocks naturally, anthropogenic activities such as the excessive application of agrochemicals, sewage sludge, wastewater, biosolids, and manure have significantly expanded their accumulation in agricultural soils [9,10]. Heavy metals persist in soils, accumulate through the soil–plant interface, and pose serious threat to plant productivity, food safety, ecosystem health, and human well-being [11,12,13]. It is estimated that nearly 10 million people worldwide are directly affected by HM-contaminated soils [14]. Soil degradation by salinity and metal threatens long-term food security and environmental stability [15,16].

Many arid, semi-arid and coastal regions are facing both salt stress and heavy metal contamination. This combination makes it difficult for plants to survive. Since conventional crops often struggle under these conditions, it is necessary to find alternative biological solutions [17,18]. Halophytes, which are plants that thrive in salty environments, have shown great promise. They can tolerate many different stressors. While halophytes make up only about 1% of the world’s plant life and about 2% of terrestrial plant species, they can survive at salt levels that would kill almost 99% of ordinary plants [18]. Their tolerance varies according to species and populations. Key mechanisms include ion compartmentalization, osmotic adjustment, antioxidant defense and metabolic modifications [5].

It is important to note that halophytes inhabiting saline coastal and desert environments have developed adaptive mechanisms that enable them to tolerate not only high salinity but also elevated concentrations of trace metals [19]. Previous studies have shown that the resistance of halophytes to heavy metals is closely associated with their salt tolerance traits [20]. Under such combined stress conditions, plants involved multiple physiological and molecular mechanisms, including ion homeostasis, osmotic adjustment, and activation of antioxidant systems, which have been widely documented in recent genomic and biotechnological studies [21].

Due to their high biomass production, their deep rooting systems and their perennial growth habits, several halophyte species have been identified as promising candidates for phytoremediation strategies, especially under combined salinity and HM stress [18,22]. Therefore, the phytoremediation of saline soils contaminated by HM requires the identification of halophyte species capable of thriving under double stress conditions [23].

While interest in halophytes is growing, only a small number of studies have examined halophyte’s functional biology and the potential of halophytes for phytoremediation of heavy metal-contaminated saline soils [18,22]. Furthermore, numerous studies have demonstrated that a number of halophyte species are effective candidates for phytoextraction or phytostabilization of heavy metals, including *Salicornia europaea* L. [24], *Salvadora persica* L. [25], *Suaeda salsa* (L.) Pall. [26], *Halogeton glomeratus* (M.Bieb.) C.A.Mey. [27], *Suaedamonoica* (Forssk. ex J.F.Gmel.) [28], *Tamarix indica* Willd. [28], *Cressa critica* L. [28], and *Atriplex halimus* L. [29]. Nonetheless, there is a need for further exploration in search of indigenous halophytes that can be applicable for the different types of saline soils found in the Mediterranean and North African regions.

In this line, *Caroxylon vermiculatum* (L.) Akhani and Roalson (syn. *Salsola vermiculatum*), a perennial halophyte belonging to the Amaranthaceae family, is an unexplored species with great potential. This plant, which is originally distributedin arid and semi-arid areas of the Mediterranean region, as well as in North Africa and the Middle East, is adapted to saline steppes and wetlands [30]. Likewise, *Mesembryanthemum nodiflorum* L., a halophyte belonging to the Aizoaceae family, which is mainly detected in Mediterranean salt marshes, is well recognized for its high tolerance to high salinity, osmotic regulation capacityanda wide variety of bioactive secondary metabolites with antioxidant properties [31]. Despite their ecological relevance, comparative information on their physiological responses, antioxidant systems, and mineral element distribution under natural saline and metal-affected conditions remains limited.

Therefore, the present study aims to provide a comparative assessment of these two native halophytes collected from the Ariana Sebkha (Tunisia), focusing on their mineral element distribution, oxidative stress markers, antioxidant enzyme activities, and phenolic composition. Specifically, this work aims to: (i) evaluate the distribution of mineral and trace elements in plant tissues and their translocation patterns, (ii) characterize oxidative stress responses and antioxidant defense mechanisms, including enzymatic and non-enzymatic components, and (iii) assess their potential contribution to phytoremediation strategies under saline and metal-affected conditions.

By integrating physiological, biochemical, and phytochemical analyses, this study provides new insights into the adaptive strategies of native halophytes in a natural saline wetland, and contributes to the identification of species with potential relevance for sustainable phytomanagement in Mediterranean environments.

## 2. Results

### 2.1. Sediment Physicochemical Characteristics

The physicochemical characteristics of soils associated with *Caroxylon vermiculatum* and *Mesembryanthemum nodiflorum* revealed marked differences in ionic composition, salinity, and nutrient status, which are likely to influence plant adaptive responses. The soil of *C. vermiculatum* exhibited higher sodium concentration (246.89 µg g^−1^), along with elevated salinity (3.21 psu) and electrical conductivity (7.1 mS cm^−1^), indicating a more saline and sodium-dominated environment compared to the *Mesembryanthemum* site (159.83 µg g^−1^ Na; 1.04 psu; 2.47 mS cm^−1^). In contrast, the soil associated with *M. nodiflorum* showed higher concentrations of calcium (470.51 mg kg^−1^), magnesium (62.98 µg g^−1^), and potassium (67.76 µg g^−1^) than that of *C. vermiculatum* (Ca: 339.79 µg g^−1^; Mg: 57.44 µg g^−1^; K: 51.99 µg g^−1^) (Table 1).

Both soils exhibited slightly alkaline pH values (7.5–7.71). Differences were also observed in nitrogen forms, with higher nitrate and nitrite contents in *C. vermiculatum* soils, whereas ammonium predominated in *M. nodiflorum* soils. Additionally, the *Mesembryanthemum* site showed higher levels of organic phosphorus (91.9 µg g^−1^) and organic matter content (3.03%) compared to *C. vermiculatum* (15.7 µg g^−1^; 2.13%), indicating a more favorable nutrient status.

Regarding trace elements, soils from the *Mesembryanthemum* site contained higher Fe (220.89 µg g^−1^) and slightly higher Cd (0.0401 µg g^−1^) compared to the *Caroxylon* site (Fe: 158.96 µg g^−1^; Cd: 0.0357 µg g^−1^), while Zn concentrations were similar between sites (~0.236 µg g^−1^) (Table 1).

### 2.2. Plant’s Mineral Element Distribution

Significant differences in macro- and microelements concentrations were observed between the two species (*p* < 0.05). *Mesembryanthemum nodiflorum* exhibited higher concentrations of Na and K in leaves compared to *Caroxylon vermiculatum*, reaching 255.34 µg g^−1^ DW and 134.39 µg g^−1^ DW, respectively, versus 132.01 µg g^−1^ DW (Na) and 109.34 µg g^−1^ DW (K) in *C. vermiculatum* (Table 2).

In addition, *M. nodiflorum* showed lower concentrations of Fe and Ca in leaves (0.39 and 7.85 µg g^−1^ DW, respectively) compared to *C. vermiculatum*, which exhibited higher levels (1.40 and 16.97 µg g^−1^ DW, respectively). Zinc concentrations were slightly higher in *M. nodiflorum* (0.06 µg g^−1^ DW) than in *C. vermiculatum* (0.03 µg g^−1^ DW), while cadmium levels remained low and comparable between both species (Table 2).

A similar trend was observed in roots, where *M. nodiflorum* accumulated higher Na and K concentrations (204.32 and 87.62 µg g^−1^ DW, respectively) than *C. vermiculatum* (115.31 and 56.86 µg g^−1^ DW). Conversely, *C. vermiculatum* exhibited higher Fe accumulation in leaves but lower levels in roots compared to *M. nodiflorum*. Calcium was predominantly accumulated in the roots of *M. nodiflorum* (27.13 µg g^−1^ DW), whereas *C. vermiculatum* showed lower root Ca content (3.59 µg g^−1^ DW). For trace elements, Zn and Cd concentrations remained relatively low in both species, with slightly higher Cd accumulation in the roots than in the leaves (Table 2).

### 2.3. Phytoremediation Indices (TF, BAF, and BCF)

The analysis of accumulation and translocation indices revealed distinct patterns between the two studied species. In *M. nodiflorum*, bioaccumulation factor (BAF) values were highest for Na (≈1.60) and K (≈1.90), while remaining markedly lower for Ca, Fe, Zn, and Cd. Similarly, bioconcentration factor (BCF) values exceeded 1 for Na and K, but were low for trace elements, indicating limited root accumulation of metals (Table 3).

In contrast, *C. vermiculatum* exhibited BAF values generally below 1 for most elements, except for K (≈2.1), suggesting restricted uptake from soil. However, translocation factor (TF) values were consistently higher than 1 for several elements (Table 3).

Variations were observed between *M. nodiflorum* and *C. vermiculatum* (Table 3). In *M. nodiflorum*, translocation factors (TFs) for Na(1.25) and K(1.53) were significantly higher than unity (*p* < 0.05), indicating preferential accumulation in aerial tissues. Conversely, Fe (TF = 0.36) and Cd (TF = 0.40) exhibited significantly lower TF values (*p* < 0.05), reflecting dominant retention in roots.

In *C. vermiculatum*, TF values calculated for leaf/root ratios were significantly higher for most elements, particularly Ca (TF = 4.72) and Fe (TF = 3.23) (*p* < 0.05). Moderate but significant TF values were also recorded for Na, K, Zn, and Cd (*p* < 0.05) (Table 3).

### 2.4. Photosynthetic Pigments and Primary Metabolites

Photosynthetic pigment contents differed significantly between species (*p* < 0.05). *C. vermiculatum* exhibited higher chlorophyll a content and a significantly greater Chl a/Chl b ratio (2. 35), whereas *M. nodiflorum* showed higher carotenoid contents (2.20 mg g^−1^, *p* < 0.05) (Table 4).

Soluble sugar concentrations were significantly higher in *C. vermiculatum* leaves (49. 36 µmol g^−1^ DW) compared to *M. nodiflorum* with registered value of 45.59 µmol g^−1^ DW (*p* < 0.05), while total protein contents did not show significant differences between species with a similar value of 0.75 mg pro g^−1^ FW (Table 4).

### 2.5. Oxidative Stress Markers

Oxidative stress markers varied significantly between the two species (Table 5). *M. nodiflorum* leaves exhibited significantly higher hydrogen peroxide (H_2_O_2_) and malondialdehyde (MDA)levels (2.25 µmol g^−1^ FW and 7.46 nmol g^−1^ FW, respectively) compared to *C. vermiculatum* (*p* < 0.05). In contrast, *C. vermiculatum* maintained significantly lower H_2_O_2_ (1. 33 µmol g^−1^ FW) and MDA concentrations (5.23 nmol g^−1^ FW) (*p* < 0.05) (Table 5).

### 2.6. Antioxidant Capacity and Enzymatic Activities

The total antioxidant capacity, measured by the reducing power assay and DPPH radical scavenging activity was significantly higher in *C. vermiculatum* than in *M. nodiflorum* (*p* < 0.05) (Table 5).

Regarding antioxidant enzymes, *C. vermiculatum* showed significantly higher activities of superoxide dismutase (SOD) (*p* < 0.05), whereas guaiacol peroxidase (GPX) activity and Catalase (CAT) activity did not show significant differences between species (*p* > 0.05) (Table 6).

### 2.7. Phenolic Compounds and Non-Enzymatic Antioxidants

Quantitative analysis revealed significant differences in phenolic compounds between species (Table 6). *C. vermiculatum* exhibited higher total phenolic content (0.392 mg GAE g^−1^ DW), flavonoid content (0.150 mg QE g^−1^ DW), and proanthocyanidines (1.147 mg CE g^−1^ MS) compared with *M. nodiflorum* (0.367, 0.137, and 0.966 mg g^−1^ DW, respectively; *p* < 0.05) (Table 7).

**Figure 1:**
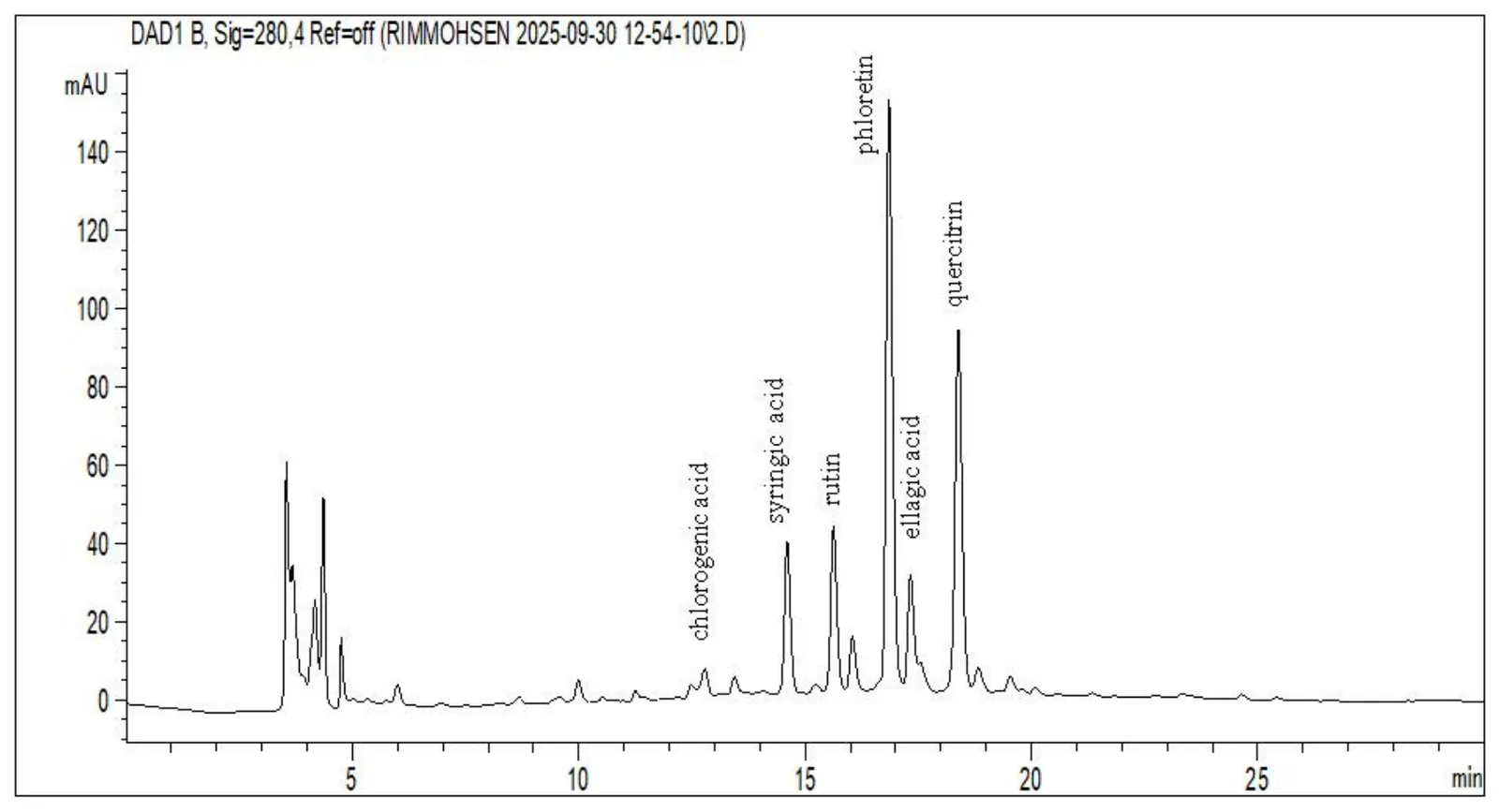
HPLC chromatogram of phenolics compounds and their derivatives from *Caroxylon vermiculatum*.

HPLC analysis revealed marked differences in phenolic composition between species. *C. vermiculatum* presented a higher diversity and abundance of phenolic compounds, including (4.23 ± 0.24 mg g^−1^ DW), quercitrin (1.06 ± 0.09 mg g^−1^ DW), and rutin (0.905 ± 0.07 mg g^−1^ DW), ellagic acid (0.313 ± 0.06 mg g^−1^ DW), chlorogenic acid (0.085 ± 0.006 mg g^−1^ DW), and syringic acid (0.085 ± 0.005 mg g^−1^ DW), this profile was associated with substantially elevated total polyphenol content values (*p* < 0.05) (Table 7 and Figure 1).

**Figure 2:**
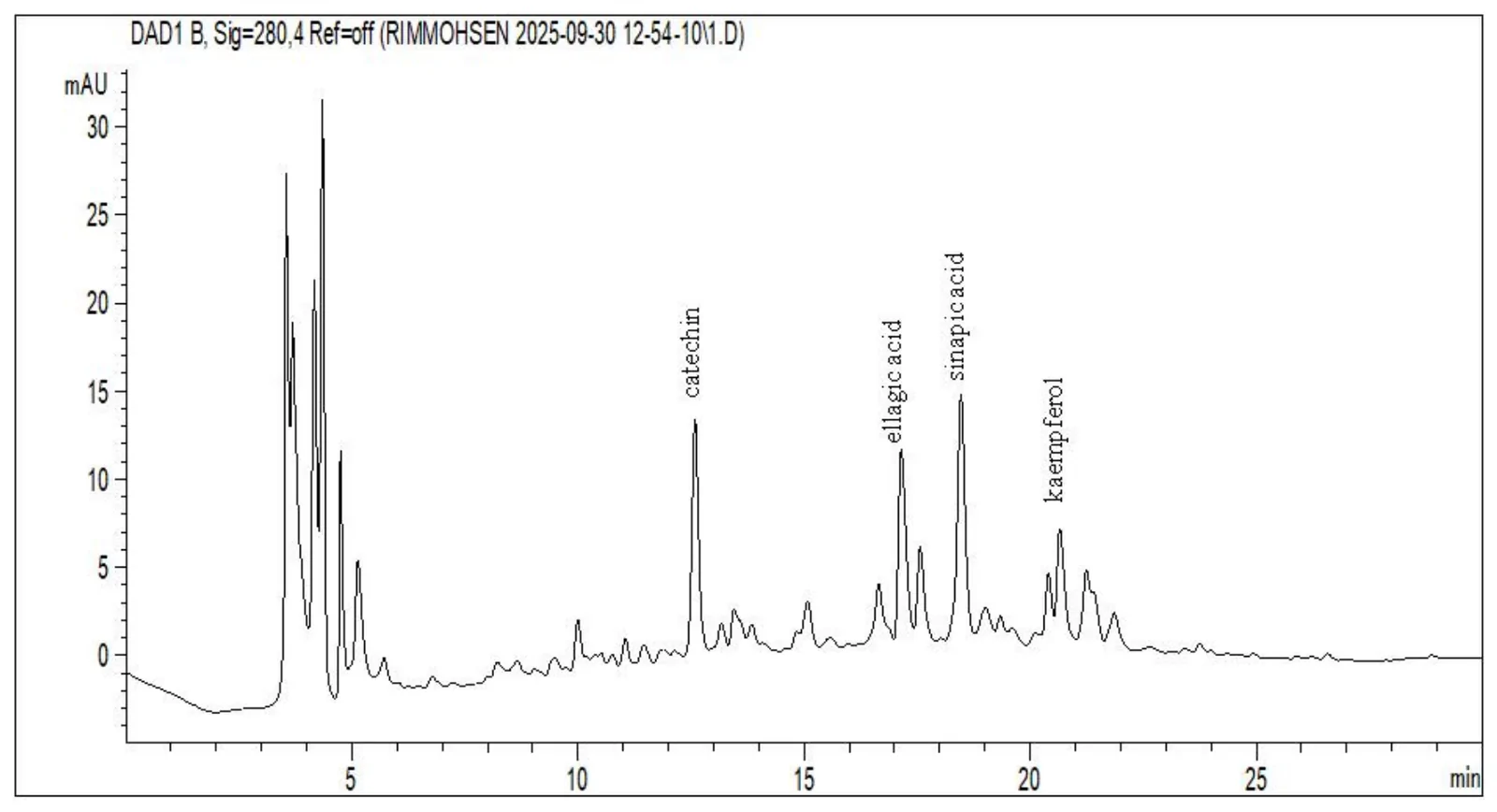
HPLC chromatogram of phenolics compounds and their derivatives from *Mesembryanthemum nodiflorum*.

In contrast, *M. nodiflorum* showed a restricted phenolic profile dominated by sinapic acid 1.62 ± 0.1 mg g^−1^ DW) and catechin (0.36 ± 0.03 mg g^−1^ DW), with significantly lower overall phenolic diversity (*p* < 0.05) (Table 7 and Figure 2).

### 2.8. Multivariate Analysis of Biochemical and Antioxidant Parameters

**Figure 3:**
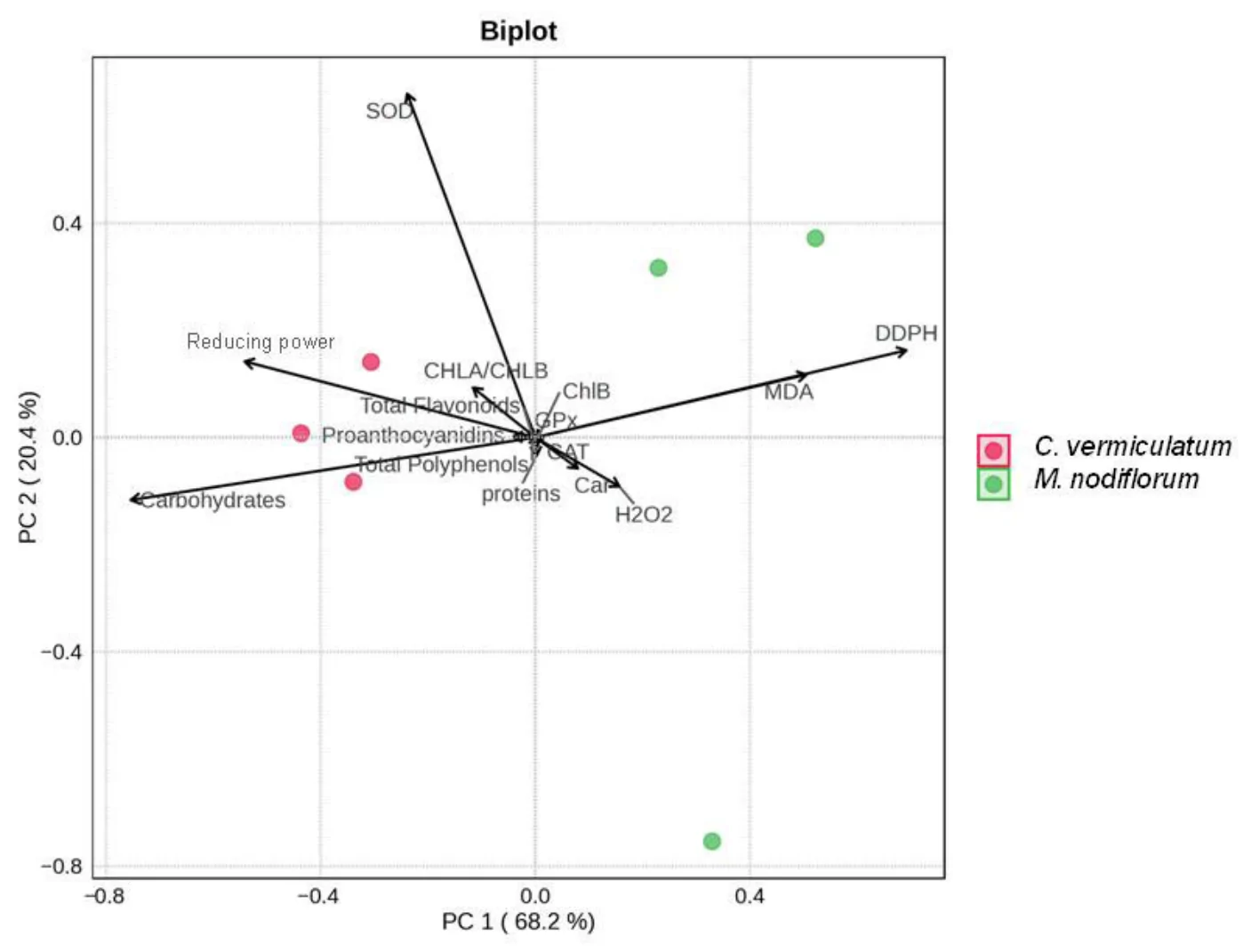
Principal component analysis (PCA) showing the relationships between biochemical parameters, antioxidant activities, and the two halophyte species.

Principal component analysis (PCA) was applied to investigate the relationships among biochemical parameters, antioxidant activities, and oxidative stress markers in the two halophyte species (Figure 3). The first two principal components explained 88.6% of the total variance, with PC1 accounting for 68.2% and PC2 explaining 20.4%, indicating that these axes adequately summarized the variability of the dataset.

The PCA score plot revealed a clear separation between the two species along PC1, reflecting distinct biochemical characteristics. *Caroxylon* samples were positioned on the negative side of PC1, while *Mesembryanthemum* samples were distributed on the positive side, highlighting strong differences in their metabolic profiles (Figure 3).

The loading plot indicated that Caroxylon was strongly associated with phenolic compounds, flavonoids, proanthocyanidins, carbohydrates, and reducing power. This suggests that the antioxidant capacity of this species is mainly linked to the accumulation of secondary metabolites with reducing properties.

Conversely, *Mesembryanthemum* samples were positively correlated with DPPH. radical scavenging activity, malondialdehyde (MDA), hydrogen peroxide (H_2_O_2_), guaiacol peroxidase (GPX), and calcium (Ca). These variables are commonly related to oxidative stress and enzymatic antioxidant responses, indicating that this species may rely more strongly on enzymatic defense mechanisms (Figure 3).

The second principal component (PC2) was mainly influenced by superoxide dismutase (SOD) and catalase (CAT) activities, which represent key enzymes involved in the detoxification of reactive oxygen species (Figure 3).

**Figure 4:**
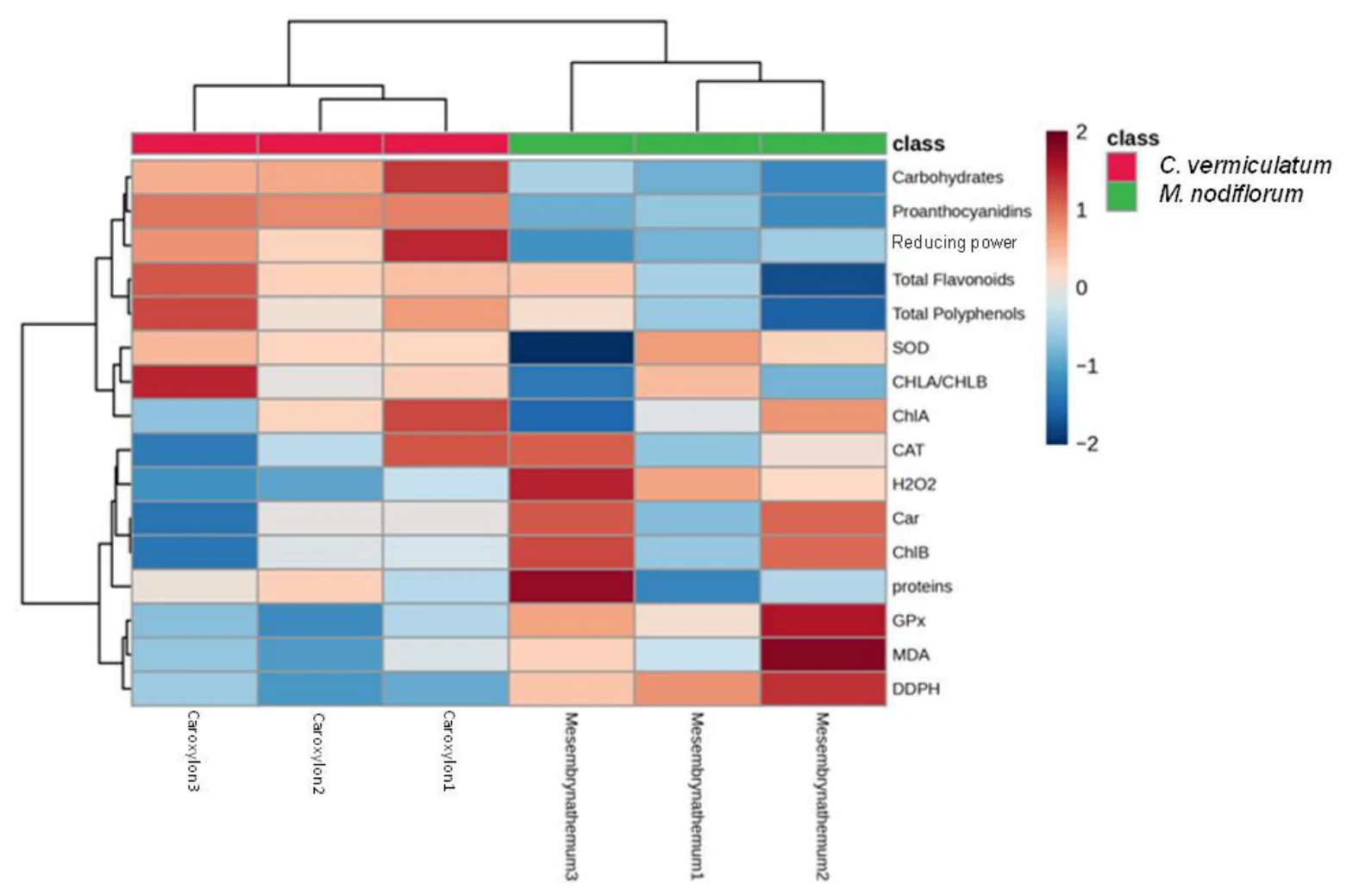
Heatmap with hierarchical clustering illustrating the distribution of biochemical parameters and antioxidant activities in the two halophyte species.

To further explore the relationships among variables and samples, hierarchical clustering analysis combined with heatmap visualization was performed. The dendrogram clearly separated the samples into two distinct clusters corresponding to the two plant species, confirming the discrimination observed in the PCA (Figure 4).

The heatmap revealed that *Caroxylon* samples were characterized by higher levels of phenolic compounds, flavonoids, proanthocyanidins, carbohydrates, and reducing power activity, whereas *Mesembryanthemum* samples exhibited relatively higher levels of DPPH. radical scavenging activity, oxidative stress indicators (MDA and H_2_O_2_), and antioxidant enzyme activities such as GPX (Figure 4).

## 3. Discussion

Salinity and heavy metal contamination frequently co-occur in arid, semi-arid, and coastal ecosystems, posing severe constraints on plant growth, soil fertility, and ecosystem functioning [32,33]. The present study provides the first comparative assessment of phytoremediation-related traits of *C. vermiculatum* and *M. nodiflorum.* The latter are two native halophytes from saline wetlands, focusing on mineral accumulation, photosynthetic performance, primary metabolism, and oxidative stress responses which are key determinants of plant fitness and indicative phytoremediation potential under saline-metals conditions [34,35].

### 3.1. Sediment Conditions as Drivers of Plant Adaptive Strategies

The observed differences in sediment physicochemical properties clearly indicate that edaphic conditions play a central role in shaping the adaptive strategies of the two halophyte species. The *C. vermiculatum* site was characterized by higher salinity, sodium dominance, and nitrate prevalence, suggesting relatively oxidizing conditions that may favor nitrate assimilation and influence plant metabolic pathways, including the synthesis of protective secondary metabolites [36]. In contrast, sediments associated with *M. nodiflorum* were enriched in organic matter, ammonium, phosphorus, and divalent cations (Ca and Mg), reflecting a more reduced and nutrient-rich environment that likely enhances microbial activity, nutrient turnover, and potentially metal mobility [37].

These contrasting sediment conditions are consistent with the distinct physiological responses observed between the two species. The relatively balanced ionic composition and higher nutrient availability in the *Mesembryanthemum* habitat may facilitate ion uptake and accumulation, supporting its salt-accumulating strategy and higher translocation of Na^+^ to aerial tissues [38]. Conversely, the more saline and sodium-dominated environment of *Caroxylon* appears to select for a more conservative strategy based on controlled ion uptake and internal regulation, limiting excessive accumulation while maintaining ionic homeostasis [39].

Furthermore, differences in redox conditions and nutrient forms (nitrate vs.ammonium) may indirectly influence metal speciation and availability, thereby affecting plant uptake patterns and oxidative responses [40]. The higher organic matter content in the *Mesembryanthemum* site may increase the complexation and mobility of certain trace elements, whereas the more mineral and saline environment of *Caroxylon* may restrict their bioavailability [41].

Overall, these findings highlight that sediment characteristics, including salinity, nutrient forms, and ionic balance, act as key environmental drivers of plant functional strategies, modulating both ion transport mechanisms and stress responses [42].

### 3.2. Mineral Translocation and Ion Management Strategies

The higher accumulation of Na and K observed in *M. nodiflorum,* particularly in leaves, indicates a salt-inclusion strategy typical of succulent halophytes. In such species, Na^+^ ions are actively transported to aerial tissues and compartmentalized within vacuoles through tonoplast Na^+^/H^+^ antiporters, thereby maintaining low cytosolic Na concentrations while contributing to osmotic adjustment. This mechanism allows plants to maintain water uptake and cell turgor under saline conditions. Similar ion compartmentalization strategies have been reported in halophytes such as *Salicornia europaea* [24], *Suaeda maritima* [26], and *Atriplex halimus* [29].

Maintaining potassium homeostasis is another key determinant of salt tolerance, since K is required for enzyme activation, stomatal regulation, and protein synthesis [3,20]. The ability of *M. nodiflorum* to maintain relatively high K levels despite elevated Na concentrations suggests the presence of selective ion transport systems that preserve cytosolic K^+^/Na^+^ balance [43,44], a mechanism commonly reported in tolerant halophytes like *Aeluropus littoralis* [45] and *Limonium species* [46].

In contrast, the lower Na accumulation observed in *C. vermiculatum* suggests a salt-exclusion or ion-regulation strategy. In fact, Selective Na^+^ transport, as demonstrated by specific transporters such as ZmHAK17, may also operate in halophytes to regulate Na^+^ loading and distribution between roots and shoots [47]. Similar strategies have been described in halophytes such as *Salsola* [48] and *Tamarix* [49] species. In this mechanism, Na uptake is restricted at the root level through selective transport barriers and reduced xylem loading, thereby preventing excessive ionic toxicity in photosynthetic tissues [50,51]. The relatively higher retention of ions in the root system observed in *C. vermiculatum* further supports the role of roots as a regulatory barrier controlling ion transport to shoots [43,52], which may still contribute to phytoremediation through phytostabilization and controlled ion sequestration.

These contrasting strategies are further supported by bioaccumulation (BAF) and bioconcentration (BCF) factors. *M. nodiflorum* exhibited BAF and BCF values greater than 1 for major cations such as Na and K, indicating an efficient uptake and accumulation from the surrounding environment. In contrast, *C. vermiculatum* showed generally lower BAF and BCF values for most elements, reflecting restricted uptake and supporting its ion-exclusion strategy. These results confirm that the relatively moderate element concentrations observed in plant tissues are not indicative of weak tolerance, but rather of species-specific regulation mechanisms, where ion homeostasis is tightly controlled rather than maximized. Metal exclusion and chelation strategies, well characterized in aluminum stress tolerance, may similarly contribute to limiting the uptake and translocation of other metals such as Cd and Zn in halophytic species [53]. Halophytes exhibit diverse adaptive mechanisms to cope with saline environments, ranging from extreme ion accumulation to selective ion regulation [1,23].

The variations in mineral translocation patterns were further highlighted by translocation factor (TF) analysis. The high TF values for Na and K in *M. nodiflorum* are characteristic of salt-accumulating halophytes as reported in *Salicornia* [24] and *Suaeda* [26], where Na is preferentially transported to shoots and stored in aerial tissues as part of the tolerance strategy. In contrast, lower TF values for Fe and Cd suggest preferential root retention, a mechanism commonly observed in halophytes such as *Atriplex halimus* [29] and *Spartina alterniflora* [54], which limits the translocation of potentially toxic elements to photosynthetic tissues.

In *C. vermiculatum*, relatively high TF values for Ca and Fe indicate enhanced upward transport of essential nutrients, consistent with observations in halophytes such as *Limonium sinuatum* and *Plantago maritima* [54,55]. Calcium plays a key role in maintaining membrane stability under stress, while iron is essential for photosynthetic processes, suggesting that selective translocation of these elements contributes to maintaining physiological performance under saline conditions [20].

Finally, the low translocation observed in both species is consistent with findings reported in other halophytes, where high-salinity conditions may exert an antagonistic effect on heavy metal uptake through ionic competition at root absorption sites, as well as through dilution effects associated with enhanced growth [56]. In addition, tolerance to metal stress in many plant systems is often associated with restricted translocation and enhanced retention of toxic elements in roots, thereby limiting their accumulation in aerial tissues and reducing potential damage to photosynthetic organs [57].

### 3.3. Photosynthetic Performance and Primary Metabolism

The composition of the photosynthetic pigments differed significantly between the two species, presenting distinct adaptation strategies to environmental stress. The elevated chlorophyll a content and increased Chl a/Chl b ratio observed in *C. vermiculatum* suggesting potential differences in photosynthetic acclimation under stress conditions [58,59]. In fact, an augmentation in Chl a/Chl b ratio is often associated with optimized light harvesting and reduced photoinhibition, allowing plants to maintain carbon assimilation in saline environments [60].under stressful environmental conditions. Such adjustments are considered important adaptive responses in halophytes, as salinity and heavy metals can impair photosynthetic machinery through ionic imbalance and oxidative damage [61].

On the other hand, the results show that there was a high concentration of chlorophyll b and carotenoids in *M. nodiflorum*, reflecting the succulence character of halophytes as reported in previous studies related to *Salicornia europaea*, *Suaeda maritima*, and *Sesuvium portulacastrum* [54]. Carotenoids are known to be essential in dissipating excess energy in these succulent halophytes like *Salicornia europaea*, as they help protect photosynthetic appratus against ROSwithin chloroplasts. Through mechanisms such as the xanthophyll cycle, carotenoids convert excess absorbed light energy into heat, thereby limiting photooxidative damage damage [19,62].

The significant concentration of soluble sugars detected in the leaves of *C. vermiculatum* implies a crucial role for carbohydrates in osmotic protection, membrane stabilization, and metabolism. Likewise, similar sugar content has been determined in other halophytes like *Aeluropus littoralis* and *Plantago maritima* [63,64]. Soluble sugars function not only as osmolytes that contribute to cellular osmotic balance but also as signaling molecules involved in stress responses and redox regulation. Additionally, sugars can stabilize cellular membranes and proteins while serving as substrates for respiration and metabolic repair processes under stress conditions [63,65].

The absence of significant differences in total protein levels between the two species implies that stress tolerance depends more on metabolic reprogramming and enzyme activity modulation than on large-scale changes in protein accumulation. Under saline and metal stress, plants often adjust metabolic pathways to optimize energy use and maintain essential cellular functions rather than increasing total protein synthesis [66].

Thus, the effect of heavy metal stress has been observed to negatively influence photosynthesis by interfering with chlorophyll biosynthesis and damaging chloroplast structures. Metals may replace Mg^2+^ in the chlorophyll molecule or stimulate chlorophyllase activity, leading to pigment degradation. In addition, heavy metal-induced ROS production can damage photosynthetic membranes and reduce photosynthetic efficiency [59]. Therefore, the variations observed in pigment composition and soluble sugar levels between the two species likely reflect different strategies to maintain photosynthetic stability and metabolic balance under combined salinity and metal stress [67].

### 3.4. Oxidative Stress and Antioxidant Enzyme Responses

Oxidative stress markers revealed pronounced interspecific differences between *M. nodiflorum* and *C. vermiculatum*. The significantly higher H_2_O_2_ and MDA levels detected in *Mesembryanthemum* indicate enhanced oxidative pressure and lipid peroxidation, processes commonly associated with ionic toxicity and metabolic imbalance under salinity and metal stress [68,69]. MDA is widely recognized as a reliable indicator of membrane damage and oxidative stress intensity in plants exposed to adverse environmental conditions, including salinity and heavy metals [69]. Elevated ROS production is commonly associated with high salt accumulation in aerial tissues, which can disrupt electron transport chains in chloroplasts and mitochondria and lead to the formation of superoxide radicals and hydrogen peroxide [69]. Similar increases in H_2_O_2_ and MDA have been reported in salt-accumulating halophytes such as *Salicornia europaea, Suaeda maritima*, and *Sesuvium portulacastrum*, where high Na^+^ sequestration in aerial tissues often results in elevated ROS production and enhanced lipid peroxidation [1,54].

In contrast, *C. vermiculatum* showed significantly lower H_2_O_2_and MDA concentrations, presenting higher membrane stability and more effective control of ROS accumulation. The same oxidative restraint has been observed in ion-excluding or nutrient prioritizing halophytes like *Atriplex halimus*, *Limonium* spp., and *Tamarix* spp., which reduce excessive Na^+^ translocation, causing a minimalized oxidative damage in photosynthetically active tissues [44,70]. This reduced oxidative burden suggests the presence of efficient antioxidant systems and/or lower stress-induced ROS production [35,71]. Such traits are advantageous for long-term survival in harsh environments and are particularly relevant for phytostabilization purposes, where sustained plant cover is essential [72].

The activity patterns of antioxidant enzymes provide further insight into the oxidative stress responses of the two halophyte species. In plants, superoxide dismutase (SOD) represents the first line of defense against oxidative stress by catalyzing the dismutation of superoxide radicals (O_2_^−^) into hydrogen peroxide (H_2_O_2_). The resulting H_2_O_2_ is subsequently detoxified by enzymes such as catalase (CAT) or peroxidases to prevent oxidative damage to cellular components [73,74]. In the present study, SOD activity was significantly higher in *C. vermiculatum*, suggesting a more efficient initial conversion of superoxide radicals to water and oxygen [35,75]. In contrast, no significant differences were observed in CAT and GPX activities between the two species. This indicates that the downstream detoxification of H_2_O_2_ may occur at comparable rates in both plants, despite differences in upstream ROS production. The higher SOD activity in *C. vermiculatum* may therefore contribute to improved control of oxidative stress by limiting the accumulation of highly reactive superoxide radicals, thereby reducing potential damage to cellular membranes and metabolic processes.

Similar enzyme patterns have been observed in stress-tolerant halophytes like *Aeluropus littoralis* and *Plantago maritima* [19]. The capacity to efficiently regulate ROS production and scavenging is a critical determinant of stress tolerance and may influence the ecological adaptation and phytoremediation potential of these halophytic species in saline and metal-contaminated environments. These antioxidant responses are part of complex regulatory networks involving transcription factors and hormone-mediated signaling pathways that coordinate plant adaptation to multiple environmental stresses [76].

### 3.5. Role of Phenolic Compounds in Stress Tolerance

Phenolic compounds represent an important component of the non-enzymatic antioxidant defense system in plants exposed to environmental stress. In the present study, *C. vermiculatum* showed a higher diversity and abundance of phenolic compounds, including flavonoids and phenolic acids such as phloretin, rutin, quercitrin, ellagic acid, and chlorogenic acid. These compounds are well known for their capacity to scavenge reactive oxygen species (ROS), stabilize cellular membranes, and chelate metal ions, thereby reducing oxidative damage induced by salinity and heavy metals. The antioxidant activity of phenolics is largely attributed to their hydroxyl groups, which can donate electrons or hydrogen atoms to neutralize free radicals [77]. In addition, phenolic compounds are capable of forming stable complexes with transition metals such as Fe and Cu, thereby limiting metal-catalyzed Fenton reactions that generate highly reactive hydroxyl radicals. Through these mechanisms, phenolics contribute not only to ROS detoxification but also to the prevention of oxidative chain reactions that damage cellular lipids, proteins, and nucleic acids [78].

The higher total polyphenol content and stronger reducing power observed in *C. vermiculatum* support the idea that secondary metabolites play a major role in maintaining redox balance in this species [79]. Similar relationships between phenolic accumulation and oxidative stress tolerance have been reported in halophytes such as *Atriplex halimus*, *Limonium* spp., and *Tamarix* spp., where flavonoids and phenolic acids constitute a major protective mechanism against salinity-induced oxidative stress [80].

In contrast, *M. nodiflorum* displayed a more limited diversity of phenolic compounds, with sinapic acid and catechin being the dominant metabolites. Although these compounds possess antioxidant activity, a reduced diversity and concentration of phenolics may limit the efficiency of the non-enzymatic antioxidant system. This could partly explain the higher levels of oxidative stress markers (H_2_O_2_ and MDA) observed in this species. Similar patterns have been reported in salt-accumulating halophytes such as *Salicornia europaea* and *Suaeda salsa*, where oxidative stress mitigation relies more heavily on enzymatic antioxidants and pigment-based photoprotective mechanisms [54].

Multivariate analysis further supports these contrasting biochemical strategies. The strong association between *C. vermiculatum* and phenolic compounds, flavonoids, and proanthocyanidins suggests that its antioxidant defense relies primarily on the accumulation of secondary metabolites with strong radical-scavenging capacity. Conversely, the correlation observed between *M. nodiflorum*, oxidative stress markers, and antioxidant enzyme activity indicates a defense strategy more dependent on enzymatic detoxification systems. Overall, the multivariate analyses demonstrate the presence of distinct biochemical and antioxidant profiles between the two halophyte species, suggesting different strategies for coping with oxidative stress in saline environments.

Previous studies have reported that phenolics are a vast antioxidant pool in halophytes, acting successfully both as ROS scavengers and secondary non-enzymatic antioxidants when the primary antioxidant system is saturated or partially rendered ineffective due to persistent stress [81,82]. Flavonoids, for instance, also possess a secondary role in ROS scavenging, acting in conjunction with antioxidant enzymes to safeguard cells from environmental stresses [83,84].

Overall, these results highlight the complementary roles of enzymatic and non-enzymatic antioxidants in halophyte stress tolerance. While enzymatic systems provide rapid detoxification of ROS, phenolic compounds offer additional protection through radical scavenging, membrane stabilization, and metal chelation [84].

### 3.6. Implications for Phytoremediation and Phytomanagement

The contrasting physiological and biochemical responses observed between *M. nodiflorum* and *C. vermiculatum* provide important insights into their potential roles in phytomanagement of saline and metal-affected environments. Phytoremediation efficiency depends not only on metal accumulation, but also on plant tolerance to stress, maintenance of metabolic activity, and the ability to persist under harsh environmental conditions.

The relatively higher accumulation and translocation of Na in *M. nodiflorum* indicate a salt-inclusion strategy typical of succulent halophytes. In this species, ions are transported to aerial tissues and compartmentalized within vacuoles, contributing to osmotic adjustment under saline conditions. Although this capacity suggests a potential contribution to ion removal, the relatively moderate BAF and BCF values observed for trace metals indicate that this species does not behave as a strong hyperaccumulator. Therefore, its role in phytoremediation is more likely associated with ion regulation and partial extraction of soluble salts rather than efficient heavy metal phytoextraction.

In addition, the elevated oxidative stress markers detected in *M. nodiflorum* suggest that this species operates under higher physiological constraints, which may limit its long-term performance under severe or prolonged stress conditions. Sustained growth and physiological stability are key requirements for effective phytoremediation, and oxidative imbalance may reduce remediation efficiency over time.

In contrast, *C. vermiculatum* exhibited traits more consistent with phytostabilization strategies, including controlled ion uptake, lower translocation of potentially toxic elements, reduced oxidative damage, and efficient antioxidant defenses. These characteristics favor the maintenance of cellular homeostasis and contribute to limiting the transfer of contaminants to aerial tissues. The relatively higher root retention (BCF) observed for some elements supports its potential role in immobilizing metals in the rhizosphere, thereby reducing their mobility and ecological risk.

Furthermore, the enhanced accumulation of phenolic compounds in *C. vermiculatum* may contribute to metal chelation and ROS scavenging, reinforcing its capacity to cope with combined salinity and metal stress. Such mechanisms are known to reduce metal bioavailability and protect cellular structures under adverse conditions.

Overall, the present findings suggest that both species contribute to phytomanagement primarily through complementary mechanisms. *M. nodiflorum* appears to rely on ion accumulation and osmotic adjustment, while *C. vermiculatum* exhibits stronger regulation of ion transport and oxidative balance. However, given the moderate accumulation levels and the absence of biomass-based removal estimates, the application of these species should be considered mainly in the context of phytostabilization and ecological restoration rather than intensive phytoextraction. The use of native halophytes with distinct adaptive strategies remains a promising approach for the sustainable management of saline and moderately contaminated environments.

## 4. Materials and Methods

### 4.1. Study Area Description

**Figure 5:**
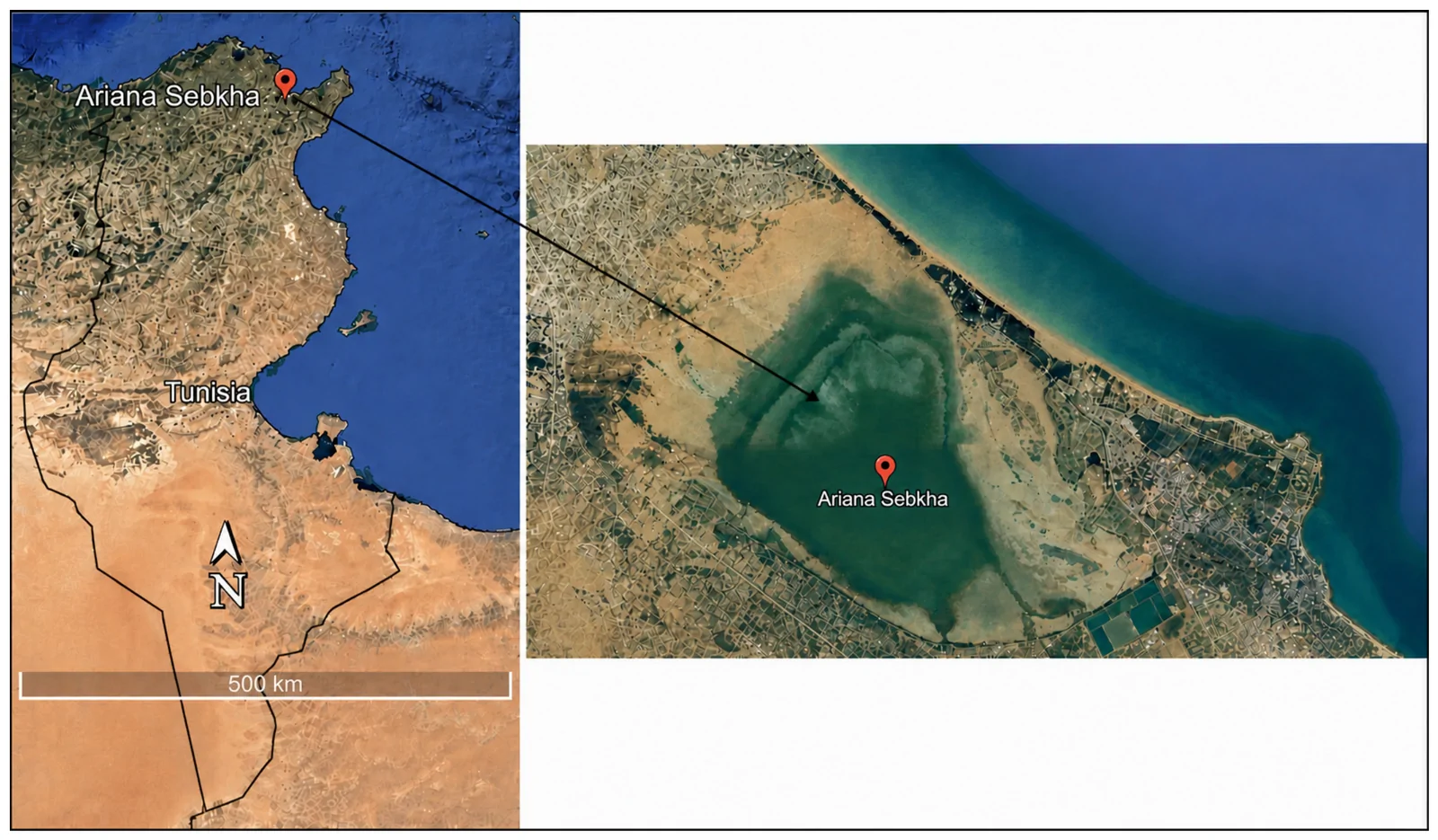
Map showing the Ariana sebkha sites.

The study area was the Ariana Sebkha (saline wetland) which is a basin of 5000 hectares located north of Lake Tunisia, from which it is separated by the plain from the Soukra (Figure 5). A coastal dune cordon extending between Raoued and Gammarth also separates it from the Gulf of Tunis. It communicates with the sea intermittently thanks to an opening at the level of the Raoued beach [85]. Its main problems are related to its pollution by the uncontrolled discharge of effluents from drainage canals which mainly carry domestic wastewater or sometimes even wastewater discharged by Cherguia and Choutrana wastewater treatment plants. Thus, nearly 20,000 m^3^/d of untreated effluent flows directly into the sebkha, to which are added the inputs from overflows of the sludge basins of the treatment plants [86]. Nowadays, the situation is aggravated by the discharge of plluted and toxic materials into covered tanks, thus threatening the groundwater. Studies have shown that these media are contaminated by trace metal elements such as Cd, Cu, Ni and Pb [87].

### 4.2. Plant Sampling and Chemical Analysis

Mature individuals of *Caroxylon vermiculatum* and *Mesembryanthemum nodiflorum* were collected from the Ariana Sebkha during the growing season April 2025. For each species, three independent plants were randomly selected from the study site, and each plant represented a biological replicate. Shoots and roots were separated immediately after collection. Each biochemical and physiological measurement was performed in triplicate to ensure analytical reproducibility, thoroughly rinsed with distilled water, and then oven-dried at 60 °C to constant weight. The dried tissues were ground to a fine powder. All plant samples were rinsed thoroughly in distilled water to remove surface dust. For mineral analysis, approximately 0.1 g of dry powder was digested with nitric acid solution prior to mineral element determination using flame photometry (Corning, Tewksbury, MA, USA) [88], and atomic absorption spectrophotometry (Perkin Elmer, Analyst 300, Waltham, MA, USA). The limits of detection (LOD) and quantification (LOQ) for mineral and metal analysis by atomic absorption spectrometry were determined according to standard analytical procedures, based on calibration curves (3σ and 10σ criteria) and signal-to-noise ratio. Quality control was ensured using standard reference solutions and blank samples.

Mineral and trace metal concentrations were expressed as µg g^−1^ DW.

### 4.3. Soil Sampling and Analysis

Soil samples were collected, properly sealed, and transported to the laboratory for analysis. Soil pH was measured following the ISO 10390:2005 standard [89]. Briefly, a soil suspension was prepared by mixing the sample with distilled water at a ratio of 1:5 (*w*/*v*), stirred for 1 h, and then allowed to settle for at least 2 h before measurement. The pH was determined using a portable multiparameter meter (HI9828, Hanna Instruments, Woonsocket, RI, USA).

Electrical conductivity (EC) was determined according to Aydi et al. [89]. by extracting soluble salts in a 1:5 (*w*/*v*) soil-to-water ratio. After agitation and filtration, EC was measured in the extract at 25 °C and expressed as mS cm^−1^ using a multiparameter meter (Hanna Instruments, Woonsocket, RI, USA).

Soil inorganic nitrogen (NH_4_^+^ and NO_3_^−^) was analyzed according to Aydi et al. [89], while total organic nitrogen and phosphorus were determined using the Kjeldahl method.

For metal analysis, soil samples (5 g, dry weight) were subjected to acid digestion using aqua regia (HCl: HNO_3_, 3:1 *v*/*v*) following Venkateswaran et al. [90]. The digestion was carried out on a heating plate at 150 °C until dryness. All digestions were performed in triplicate. The resulting extracts were analyzed for metal concentrations using an atomic absorption spectrophotometer (AAS).

The limits of detection (LOD) and quantification (LOQ) for mineral and metal analysis by atomic absorption spectrometry were determined according to standard analytical procedures, based on calibration curves (3σ and 10σ criteria) and signal-to-noise ratio. Quality control was ensured using standard reference solutions and blank samples.

### 4.4. Phytoremediation Factors

To assess the phytoremediation potential of the studied species, including their capacity for phytoextraction and phytostabilization, three commonly used indices were determined: the bioaccumulation factor (BAF), the translocation factor (TF), and the bioconcentration factor (BCF), following the approach described by Saha et al. [91]. These indices were calculatedusing the relationshipsbelow:

- Bioaccumulation factor (BAF) = concentration of the element in shoots/concentration of the element in soil;
- Translocation factor (TF) = concentration of the element in shoots/concentration of the element in roots;
- Bioconcentration factor (BCF) = concentration of the element in roots/concentration of the element in soil.

### 4.5. Chlorophylls and Carotenoids Content

Fresh leaf samples were homogenized in 80% acetone, and chlorophyll extraction was carried out for 24 h in darkness at 4 °C [92]. The extract absorbance was determined at 470, 649, and 665 nm using a UV–visible spectrophotometer (Dual Beam 8 Auto Cell UVS-2700 (Labomed, Los Angeles, CA, USA). Chlorophyll a, b, and total carotenoid contents were calculated using Lichtenthaler’s equations [92].

### 4.6. Determination of Oxidative Stress Markers and Antioxidant Activity

#### 4.6.1. Determination of Oxidative Stress Markers

Lipid peroxidation was determined according to Hodges et al. [93]. The absorbance was read at 532 nm and 600 nm with a UV–visible spectrophotometer (Specord 210 Plus, Analytik Jena, Jena, Germany). Malondialdehyde (MDA) content was determined using the extinction coefficient of 155 mM^−1^ cm^−1^ and expressed as nmol g^−1^ FW.

Hydrogen peroxide (H_2_O_2_) content was determined according to Sergiev et al. [94]. Leaf tissues were homogenized in 0.1% trichloroacetic acid (TCA) and centrifuged at 6000 *g* for 15 min at 4 °C. The supernatant was reacted with potassium iodide (1 M) in 10 mM phosphate buffer (pH 7), and absorbance was read at 390 nm using a UV–visible spectrophotometer (Specord 210 Plus, Analytik, Jena, Germany). H_2_O_2_ contents were deduced from calibration curves prepared using known concentrations of H_2_O_2_.

#### 4.6.2. Determination of Antioxidant Activity

DPPH radical scavenging activity was evaluated following the approach of Hanato et al. [95]. Methanolic extracts were tested at final concentrations ranging from 10 to 200 µg mL^−1^. The decrease in absorbance of th purple-colored DPPH solution was measured at 517 nm after 30 min of incubation at room temperature in the dark. The percentage inhibition (PI %) of free radical DPPH was calculated as follows:

PI (%) = ((A_blank_ − A_sample_)/A_blank_) × 100,

where A_blank_ is the absorbance of the control reaction and A_sample_ is the absorbance in the presence of leaves extract. The IC_50_ value (concentration required to inhibit 50% of DPPH radicals) was determined from the regression equation.

Reducing power assay was determined using the method described by Oyaizu [96]. Absorbance was measured at 700 nm, and the EC_50_ value (extract concentration corresponding to an absorbance of 0.5) was calculated.

### 4.7. Enzymatic and Non-Enzymatic Antioxidant Systems

#### 4.7.1. Protein Extraction and Antioxidant Enzymes

Antioxidant enzymes were extracted from frozen leaf samples with polyvinylpyrrolidone (PVP) in 50 mM K-phosphate buffer (pH 7.8), containing 10 mM ethylenediaminetetra-acetic acid (EDTA), 1 mM dithiothreitol, 0.1 mM phenylmethyl-sulfonyl-fluoride (PMSF) acid and 1 mM dithiothreitol (DTT). After centrifugation at 12,000 *g* for 30 min, supernatant fractions were collected and used for different enzyme assays. Soluble protein contents were determined following the method described by Bradford [97], using bovine serum albumin (BSA) as the standard.

For Superoxide dismutase enzyme (SOD, EC.1.15.1.1), the activity was performed using the NBT photoreduction method, as described by Ellouzi et al. [98]. A single unit of Superoxide Dismutase (SOD) activity (U) was defined as the amount of enzyme required to induce a 50% inhibition of Nitroblue Tetrazolium (NBT) reduction. The absorbance was assessed at 560 nm.

Catalase (CAT, EC 1.11.1.6) activity was determined following the method of Ellouzi et al. [98], by monitoring the decomposition of H_2_O_2_, through the decrease in absorbance at 240 nm. CAT activity was expressed as U CAT mg^−1^ proteins.

Guaiacol peroxidase (GPX, EC.1.11.1.7) was assessed according to Ellouzi et al. [98]. Guaiacol oxidation (tetraguaiacol formation) was monitored at 470 nm. The increase of absorbance indicates the oxidation of guaiacol. The GPX activity was expressed as U/mg proteins.

#### 4.7.2. Determination of the Amounts of Phenolic Compounds

Total phenolic groups were determined using spectrophotometric assays to provide a global estimation of major phenolic classes. Total phenolic content was determined using the Folin–Ciocalteu reagent according to Oktay et al. [99], with modifications described by Dewanto et al. [100]. A calibration curve was constructed using gallic acid as standard at concentrations ranging from 0 to 200 µg mL^−1^. Absorbance was measured at 760 nm using a UV–Vis spectrophotometer (UV-1800, Shimadzu Corporation, Kyoto, Japan), after 90 min of incubation in the dark. Results were expressed as mg gallic acid equivalents per g dry weight (mg GAE g^−1^ DW).

Total flavonoid content was determined using the aluminum chloride colorimetric method [101]. A standard curve was prepared using quercetin at concentrations ranging from 0 to 100 µg mL^−1^, and results were expressed as mg quercetin equivalents per g dry weight (mg QE g^−1^ DW).

Proanthocyanidin content was determined using the butanol–HCl assay [102]. Catechin was used as standard for the calibration curve (0–500 µg mL^−1^). Absorbance was measured at 550 nm after cooling, and results were expressed as mg catechin equivalents per g dry weight (mg CE g^−1^ DW).

### 4.8. Determination of Phenolic by Using High-Performance Liquid Chromatography (HPLC-DAD)

HPLC-DAD analysis was performed to identify and quantify individual phenolic compounds. Fresh aerial parts of the studied halophyte species were collected, shade-dried, and subjected to methanolic Soxhlet extraction. Approximately 10 g of dried plant material was extracted with 150 mL of methanol for 6 h at the boiling temperature of the solvent (~65 °C). The extracts were then filtered and concentrated under reduced pressure prior to analysis.

The identification of phenolic compounds was carried out using an HPLC system (consisting of a vacuum degasser, an autosampler, and a binary pump with a maximum pressure of 400 bar; Agilent 1260, Agilent Technologies, Waldbronn, Germany) equipped with a reversed-phase C18 analytical column (4.6 × 100 mm, 3.5 µm; Zorbax Eclipse XDB C18).

The DAD detector was set to a scanning range of 200–400 nm. Chromatograms were monitored at specific wavelengths depending on the phenolic class: 280 nm for simple phenolic acids, 320 nm for hydroxycinnamic acids, and 360 nm for flavonoids. Column temperature was maintained at 23 °C. The injected sample volume was 2 µL, and the flow rate of the mobile phase was 0.4 mL/min.

The mobile phase consisted of solvent A (acetonitrile) and solvent B (Milli-Q water containing 0.1% formic acid). The optimized gradient elution was as follows: 0–5 min, 10–20% A; 5–10 min, 20–30% A; 10–15 min, 30–50% A; 15–20 min, 50–70% A; 20–25 min, 70–90% A; 25–30 min, 90–50% A; 30–45 min, return to initial conditions.

Quantification was performed using external calibration by constructing individual standard curves for each compound (R^2^ > 0.999), without the use of an internal standard. The concentrations of phenolic compounds were expressed as mg g^−1^ dry weight (DW), based on the corresponding calibration equations, and reported as mean ± standard deviation (n = 3).

Limits of detection (LOD) and quantification (LOQ) were estimated based on signal-to-noise ratios of 3 and 10, respectively.

### 4.9. Statistical Analysis

Statistical analysis was performed using SPSS version 21.0 (SPSS Inc., Chicago, IL, USA). The experimental samples were analyzed in three replicates (n = 3), and data were expressed as mean ± standard deviation (SD). Prior to analysis, the normality of data distribution was assessed using the Shapiro–Wilk test, and the homogeneity of variances was verified using Levene’s test. The effects of species (*Mesembryanthemum nodiflorum* and *Caroxylon vermiculatum*), plant organ (roots and leaves), and their interaction were analyzed using two-way analysis of variance (ANOVA). When significant differences were detected, mean values were compared using post-hoc multiple comparison tests at *p* ≤ 0.05. Data from chlorophyll contents, oxidative stress markers, antioxidant responses, and phenolic compounds were further explored using Principal Component Analysis (PCA) and heatmap visualization performed with R software 4.2.2.

## 5. Conclusions

This study highlights clear contrasting adaptive strategies between *Mesembryanthemum nodiflorum* and *Caroxylon vermiculatum* under naturally saline and metal-affected coastal conditions.

*Mesembryanthemum nodiflorum* showed higher accumulation of Na and K, together with increased oxidative stress markers (H_2_O_2_ and MDA) and elevated guaiacol peroxidase activity, reflecting a salt-inclusion strategy associated with enhanced reactive oxygen species (ROS) production. This species also exhibited higher translocation factors (TF > 1) for Na (~1.60) and K (~1.90), indicating efficient ion transport to aerial parts, while showing limited accumulation of trace metals as indicated by low BCF and BAF values (<1 for most elements).

In contrast, *Caroxylon vermiculatum* maintained lower levels of oxidative damage, supported by stronger antioxidant defense systems, particularly higher superoxide dismutase (SOD) activity and greater accumulation of phenolic compounds and flavonoids. This species also exhibited higher retention of trace elements in roots (BCF up to ~0.5) and lower translocation (TF < 1), suggesting more efficient ion exclusion and internal detoxification mechanisms.

Overall, both species demonstrated effective but contrasting physiological and biochemical strategies for coping with saline and metal stress, involving differences in mineral uptake (Na, K, Ca, Fe, Zn, Cd), antioxidant capacity, and stress tolerance mechanisms. Given the generally low to moderate BCF and BAF values observed for trace elements, both halophytes are better suited for phytostabilization and ion regulation processes rather than efficient phytoextraction under natural environmental conditions.

## Figures and Tables

**Table 1:** Soil characterization and macro- and microelements in the soils of both studied plants.

Properties	*Mensembranthymum* Soil	*Caroxylon* Soil
Texture	Clay soil	Clay soil
pH	7.71 ± 0.02	7.5 ± 0.04
Salinity (psu)	1.04 ± 0.01	3.21 ± 0.05
Organicnitrogen (µg/g)	123.08 ± 3.41	119.17 ± 2.51
Organicphosphorus (µg/g)	91.9 ± 1.1	15.79 ± 0.9
Ammonium (µg/g)	17.84 ± 2.1	12.09 ± 1.7
Nitrates(µg/g)	16.45 ± 1.9	21.35 ± 2.2
Nitrites (µg/g)	3.35 ± 0.3	5.86 ± 0.2
EC (mS cm^−1^)	2.47 ± 0.3	7.1 ± 0.3
Na (µg/g)	159.83 ± 12.3	246.8 9 ± 15.4
K (µg/g)	67.76 ± 6.5	51.95 ± 2.5
Ca (µg/g kg)	470.51 ± 22.4	339.79 ± 18.7
Fe (µg/g)	220.89 ± 11.5	158.96 ± 8.7
Zn (µg/g)	0.24 ± 0.04	0.24 ± 0.03
Cd (µg/g)	0.040 ± 0.002	0.036 ± 0.001

**Table 2:** Macro- and microelements concentrations (expressed as µg g^−1^ DW) of two halophytes species.

	*Mesembryanthemum nodiflorum*		*Caroxylon vermiculatum*	
	Leaves	Roots	Leaves	Roots
Na	255.33 ± 31.63 a	204.32 ± 19.09 a	132.01 ± 13.44 b	115.31 ± 11.76 b
K	134.39 ± 11.56 a	87.62 ± 9.36 b	109.32 ± 11.32 c	56.86 ± 5.34 d
Fe	0.39 ± 0.03 c	1.08 ± 0.04 b	1.4 ± 0.6 a	0.43 ± 0.05 d
Ca	7.85 ± 1.17 c	27.13 ± 3.45 a	16.97 ± 2.28 b	3.59 ± 0.67 d
Zn	0.06 ± 0.004 a	0.03 ± 0.007 bc	0.03 ± 0.002 b	0.02 ± 0.005 c
Cd	0.008 ± 0.001 b	0.02 ± 0.003 a	0.01 ± 0.003 b	0.008 ± 0.001 b

Different letters indicated a significant difference at *p* < 0.05. n = 3.

**Table 3:** Phytoremediation factors of the selected plants.

	Factors	Na	K	Ca	Fe	Zn	Cd
*Mesembryanthemum nodiflorum*	TF	1.25 ± 0.08 a	1.53 ± 0.1 a	1.10 ± 0.54 b	0.36 ± 0.02 b	2.37 ± 0.46 a	0.40 ± 0.03 b
	BAF	1.60 ± 0.11 a	1.90 ± 0.15 a	0.017 ± 0.003 a	0.002 ± 0.001 b	0.25 ± 0.03 a	0.02 ± 0.004 b
	BCF	1.3 ± 0.2 a	1.3 ± 0.14 a	0.015 ± 0.004 a	0.005 ± 0.001 a	0.11 ± 0.03 a	0.5 ± 0.09 a
*Caroxylon vermiculatum*	TF	1.14 ± 0.06 a	1.92 ± 0.2 a	4.72 ± 0.76 a	3.23 ± 0.71 a	1.63 ± 0.25 b	1.49 ± 0.06 a
	BAF	0.53 ± 0.08 b	2.10 ± 0.6 a	0.05 ± 0.01 b	0.09 ± 0.02 a	0.15 ± 0.03 b	0.33 ± 0.08 a
	BCF	0.5 ± 0.04 b	1.09 ± 0.31 a	0.011 ± 0.002 a	0.003 ± 0.001 a	0.09 ± 0.01 a	0.22 ± 0.04 b

Different letters indicated a significant difference at *p* < 0.05. n = 3.

**Table 4:** Photosynthetic pigment contents represented by three replicate.

	*Mesembryanthemum nodiflorum*	*Caroxylon vermiculatum*
Chl a (mg/g FW)	0.12 ± 0.005 a	0.13 ± 0.004 a
Chl b (mg/g FW)	0.07 ± 0.009 a	0.06 ± 0.005 a
Carotenoid (mg/g FW)	2.20 ± 0.22 a	1.83 ± 0.12 b
Chl a/Chl b	1.75 ± 0.26 a	2.32 ± 0.22 b
Proteins (mg pro g^−1^ FW)	0.756 ± 0.09 a	0.752 ± 0.002 a
Soluble Sugars (µmol g^−1^ DW)	45.58 ± 0.43 a	49.36 ± 0.54 b

Different letters indicated a significant difference at *p* < 0.05.

**Table 5:** Oxidative stress markers and Antioxidant Activity.

	*Mesembryanthemum nodiflorum*	*Caroxylon vermiculatum*
MDA (nmol/g FW)	7.46 ± 0.9 a	5.23 ± 0.42 b
H_2_O_2_ (µmol/g FW)	2.25 ± 0.18 a	1.33 ± 0.13 b
FRAP (IC50 (mg/mL)	13.84 ± 1.4 a	11.07 ± 1.2 b
DPPH (IC50 (mg/mL)	19.76 ± 1.9 a	16.35 ± 0.9 b

Different letters indicated a significant difference at *p* < 0.05. n = 3.

**Table 6:** Antioxidant Enzymes Activity expressed as U/mg proteins.

	*Mesembryanthemum nodiflorum*	*Caroxylon vermiculatum*
SOD	12.51 ± 0.6 a	13.83 ± 0.5 b
GPx	0.08 ± 0.003 a	0.04 ± 0.001 a
CAT	0.38 ± 0.04 a	0.4 ± 0.06 a

Different letters indicated a significant difference at *p* < 0.05. n = 3.

**Table 7:** Phenolics Compounds Contents in Organic Extracts of Two Halophytes species.

	*Mesembryanthemum nodiflorum*	*Caroxylon vermiculatum*
Chlorogenic acid (mg g^−1^ DW)	-	0.085 ± 0.006
Syringic acid (mg g^−1^ DW	-	0.085 ± 0.005
Catechin (mg g^−1^ DW)	0.36 ± 0.03	-
Ellagicacid (mg g^−1^ DW)	0.12 ± 0.02	0.313 ± 0.06
Rutin (mg g^−1^ DW)	-	0.905 ± 0.07
Sinapic acid (mg g^−1^ DW)	1.62 ± 0.1	-
Phloretin (mg g^−1^ DW)	-	4.23 ± 0.24
Kaempferol (mg g^−1^ DW)	0.036 ± 0.005	-
Quercitrin (mg g^−1^ DW)	-	1.06 ± 0.09
Totals Polyphenols (mg GAE/g DW)	0.37 ± 0.003 a	0.392 ± 0.002 b
totalsFlavonoids (mg QE/g DW)	0.14 ± 0.001 a	0.15 ± 0.001 a
Proanthocyanidins (mg CE/g DW)	0.96 ± 0.03 a	1.15 ± 0.07 b

Different letters indicated a significant difference at *p* < 0.05. n = 3.

## Data Availability

The original contributions presented in this study are included in the article. Further inquiries can be directed to the corresponding author.

## References

[1] Flowers T.J., Colmer T.D. (2015). Plant salt tolerance: Adaptations in halophytes. Ann. Bot..

[2] Ben Hamed K., Castagna A., Ranieri A., García-Caparrós P., Santin M., Hernandez J.A., Espin G.B. (2021). Halophyte based Mediterranean agriculture in the contexts of food insecurity and global climate change. Environ. Exp. Bot..

[3] Munns R., Tester M. (2008). Mechanisms of salinity tolerance. Annu. Rev. Plant Biol..

[4] Food and Agriculture Organization of the United Nations (FAO) (2024). Global Status of Salt-Affected Soils.

[5] Clemens S. (2006). Toxic metal accumulation, responses to exposure and mechanisms of tolerance in plants. Biochimie.

[6] Lux A., Martinka M., Vaculík M., White P.J. (2011). Root responses to cadmium in plants. J. Exp. Bot..

[7] Kumar P., Sharma P.K. (2020). Soil Salinity and Food Security in India. Front. Sustain. Food Syst..

[8] Hassani A., Azapagic A., Shokri N. (2020). Predicting long-term dynamics of soil salinity and sodicity on a global scale. Proc. Natl. Acad. Sci. USA.

[9] Rashid A., Schutte B.J., Ulery A., Deyholos M.K., Sanogo S., Lehnhoff E.A., Beck L. (2023). Heavy Metal Contamination in Agricultural Soil: Environmental Pollutants Affecting Crop Health. Agronomy.

[10] Yan A., Wang Y., Tan S.N., Yusof L.K.M., Ghosh S., Chen Z. (2020). Phytoremediation: A Promising Approach for Revegetation of Heavy Metal-Polluted Land. Front. Plant Sci..

[11] Alengebawy A., Abdelkhalek S.T., Qureshi S.R., Wang M.Q. (2021). Heavy Metals and Pesticides Toxicity in Agricultural Soil and Plants: Ecological Risks and Human Health Implications. Toxics.

[12] Das S., Sultana K.W., Ndhlala A.R., Mondal M., Chandra I. (2023). Heavy Metal Pollution in the Environment and Its Impact on Health: Exploring Green Technology for Remediation. Environ. Health Insights.

[13] Mustapha L.S., Obayomi O.V., Obayomi K.S. (2025). A comprehensive review on potential heavy metals in the environment: Persistence, bioaccumulation, ecotoxicology, and agricultural impacts. Ecol. Front..

[14] Bhat S.A., Bashir O., Haq S.A., Amin T., Rafiq A., Ali M., Américo-Pinheiro J.H.P., Sher F. (2022). Phytoremediation of heavy metals in soil and water: An eco-friendly, sustainable and multidisciplinary approach. Chemosphere.

[15] Kumar M., Kumar R., Jain V., Jain S. (2017). Differential behavior of the antioxidant system in response to salinity induced oxidative stress in salt-tolerant and salt-sensitive cultivars of *Brassica juncea* L.. Biocatal. Agric. Biotechnol..

[16] Carswell A.M., Willcock S., Blackwell M.S.A., Upadhayay H.R., Harris P., McAuliffe G., Neal A.L., Rivero M.J., Cardenas L.M., Haefele S.M. (2025). Agricultural practices can threaten soil resilience through changing feedback loops. npj Sustain. Agric..

[17] Shang C., Wang L., Tian C., Song J. (2020). Heavy metal tolerance and potential for remediation of heavy metal-contaminated saline soils for the euhalophyte *Suaeda salsa*. Plant Signal. Behav..

[18] Navarro-Torre S., Garcia-Caparrós P., Nogales A., Abreu M.M., Santos E., Cortinhas A.L., Caperta A.D. (2023). Sustainable agricultural management of saline soils in arid and semi-arid Mediterranean regions through halophytes, microbial and soil-based technologies. Environ. Exp. Bot..

[19] Hasanuzzaman M., Bhuyan M.H.M.B., Zulfiqar F., Raza A., Mohsin S.M., Mahmud J.A., Fujita M., Fotopoulos V. (2020). Reactive Oxygen Species and Antioxidant Defense in Plants under Abiotic Stress: Revisiting the Crucial Role of a Universal Defense Regulator. Antioxidants.

[20] Briat J.F., Dubos C., Gaymard F. (2015). Iron nutrition, signaling, and homeostasis in plants. Trends Plant Sci..

[21] Yuan J., Cao H., Qin W., Yang S., Zhang D., Zhu L., Song H., Zhang Q. (2025). Genomic and modern biotechnological strategies for enhancing salt tolerance in crops. New Crops.

[22] Sharma J.K., Kumar N., Singh N.P., Santal A.R. (2023). Phytoremediation technologies and their mechanism for removal of heavy metal from contaminated soil: An approach for a sustainable environment. Front. Plant Sci..

[23] Singh V.K., Singh R., Rajput V.D., Singh V.K. (2023). Halophytes for the sustainable remediation of heavy metal-contaminated sites: Recent developments and future perspectives. Chemosphere.

[24] Khanlarian M., Roshanfar M., Rashchi F., Motesharezadeh B. (2020). Phyto-extraction of zinc, lead, nickel, and cadmium from zinc leach residue by a halophyte: *Salicornia europaea*. Ecol. Eng..

[25] Patel M., Parida A.K. (2021). Salinity alleviates the arsenic toxicity in the facultative halophyte *Salvadora persica* L. by the modulations of physiological, biochemical, and ROS scavenging attributes. J. Hazard. Mater..

[26] Ibraheem F., Al-Zahrani A., Mosa A. (2022). Physiological Adaptation of Three Wild Halophytic *Suaeda* Species: Salt Tolerance Strategies and Metal Accumulation Capacity. Plants.

[27] Li B., Wang J., Yao L., Meng Y., Ma X., Si E., Ren P., Yang K., Shang X., Wang H. (2019). Halophyte *Halogeton glomeratus*, a promising candidate for phytoremediation of heavy metal-contaminated saline soils. Plant Soil.

[28] Joshi A., Kanthaliya B., Rajput V., Minkina T., Arora J. (2020). Assessment of phytoremediation capacity of three halophytes: *Suaedamonoica*, *Tamarix indica* and *Cressa critica*. BiologiaFutur..

[29] Zaghdoudi R., Sghayar S., Necib M., Dekaezmaeker P., Dailly H., Zorrig W., Abdelly C., Debez A., Lutts S. (2025). Screening for Heavy Metal-Resistant Clones in the Xero-Halophyte *Atriplex halimus* L.: A Prerequisite for Phytoremediation of Polymetallic Mining Pollution in Arid Areas. Int. J. Environ. Res..

[30] Başköse İ., Yaprak A.E. (2024). A new species record for the flora of Türkiye; *Caroxylon vermiculatum* (L.) Akhani&Roalson (Chenopodiaceae/Amaranthaceae). Trak. Univ. J. Nat. Sci..

[31] Lima A.R., Gama F., Castañeda-Loaiza V., Costa C., Schüler L.M., Santos T., Salazar M., Nunes C., Cruz R.M.S., Varela J. (2021). Nutritional and Functional Evaluation of *Inula crithmoides* and *Mesembryanthemum nodiflorum* Grown in Different Salinities for Human Consumption. Molecules.

[32] Lutts S., Lefèvre I. (2015). How can we take advantage of halophyte properties to cope with heavy metal toxicity in salt-affected areas?. Ann. Bot..

[33] Liang L., Liu W., Sun Y., Huo X., Li S., Zhou Q. (2017). Phytoremediation of heavy metal contaminated saline soils using halophytes: Current progress and future perspectives. Environ. Rev..

[34] Litalien A., Zeeb B. (2020). Curing the earth: A review of anthropogenic soil salinization and plant-based strategies for sustainable mitigation. Sci. Total Environ..

[35] Hasanuzzaman M., Fujita M. (2023). Plant Responses and Tolerance to Salt Stress: Physiological and Molecular Interventions. Int. J. Mol. Sci..

[36] Isayenkov S.V., Maathuis F.J.M. (2019). Plant salinity stress: Many unanswered questions remain. Front. Plant Sci..

[37] Zhan N., Yang H., Li J., Shi X., Yang B., Sun Y., Guo G., Cui X. (2025). Spatial Distribution Characteristics and Relationships of Salt-Based Ions and Nutrients in Old Protected Vegetable Fields. Horticulturae.

[38] Pasquale Perez M.P., Carol E., Santucci L., Idaszkin Y.L. (2024). Nutrient dynamics in wetland systems associated with hydrological and anthropogenic variations in the south of Samborombón Bay, Argentina. Sci. Total Environ..

[39] de Vicente I. (2021). Biogeochemistry of Mediterranean Wetlands: A Review about the Effects of Water-Level Fluctuations on Phosphorus Cycling and Greenhouse Gas Emissions. Water.

[40] Amin I., Rasool S., Mir M.A., Wani W., Masoodi K.Z., Ahmad P. (2021). Ion homeostasis for salinity tolerance in plants: A molecular approach. Physiol. Plant..

[41] Mansoor S., Ali A., Kour N., Bornhorst J., AlHarbi K., Rinklebe J., Abd El Moneim D., Ahmad P., Chung Y.S. (2023). Heavy Metal Induced Oxidative Stress Mitigation and ROS Scavenging in Plants. Plants.

[42] Stefanowicz A.M., Kapusta P., Zubek S., Stanek M., Woch M.W. (2020). Soil organic matter prevails over heavy metal pollution and vegetation as a factor shaping soil microbial communities at historical Zn-Pb mining sites. Chemosphere.

[43] Wu J., Wang L., Ma F., Zhao L., Huang X. (2019). The speciation and distribution characteristics of Cu in *Phragmites australis* (Cav.) Trin ex. Steudel. Plant Biol..

[44] Rostampour P., Hamidian M., Dehnavi M.M., Saeidimajd G.A. (2023). Evaluation of osmoregulation and morpho-physiological responses of *Borago officinalis* under drought and salinity stress with equal osmotic potential. Biochem. Syst. Ecol..

[45] Hashemipetroudi S., Ahmadian G., Fatemi F., Nematzadeh G., Yamchi A., Kuhlmann M. (2022). Ion content, antioxidant enzyme activity and transcriptional response under salt stress and recovery condition in the halophyte grass *Aeluropus littoralis*. BMC Res. Notes.

[46] Maaloul S., Abdellaoui R., Mahmoudi M., Bouhamda T., Bakhshandeh E., Boughalleb F. (2021). Seasonal environmental changes affect differently the physiological and biochemical responses of two *Limonium* species in Sabkha biotope. Physiol. Plant..

[47] Liang X., Wang L., Pan Y., Jing W., Wang H., Liu F., Jiang C. (2025). The natural variation in shoot Na^+^ content and salt tolerance in maize is attributed to various minor-effect variants, including an SNP located in the promoter of ZmHAK11. Plant Biotechnol. J..

[48] Rasheed A., Koyro H.W., Hameed A., Gul B. (2022). Physiological responses of the xero-halophyte *Salsola drummondii* to seasonal alterations of environmental conditions in a salt desert. Ecol. Res..

[49] Sghaier D.B., Duarte B., Bankaji I., Cacador I., Sleimi N. (2015). Growth, chlorophyll fluorescence and mineral nutrition in the halophyte *Tamarix gallica* cultivated in combined stress conditions: Arsenic and NaCl. J. Photochem. Photobiol. B Biol..

[50] Bose J., Rodrigo-Moreno A., Shabala S. (2014). Ionic homeostasis and oxidative stress tolerance in plants. Plant Cell Environ..

[51] Wu H. (2018). Plant salt tolerance and Na^+^ sensing and transport. Crop J..

[52] Hassan A., López-Gresa M., Boscaiu M., Vicente Ó. (2016). Stress tolerance mechanisms in Juncus: Responses to salinity and drought in three Juncus species adapted to different natural environments. Funct. Plant Biol..

[53] Li J., Tian J., Zhou M., Tian J., Liang C. (2025). Research progress on the physiological and molecular mechanisms underlying soybean aluminum resistance. New Crops.

[54] Rahman M., Mostofa M., Keya S., Siddiqui M., Ansary M., Das A., Rahman M., Tran L. (2021). Adaptive Mechanisms of Halophytes and Their Potential in Improving Salinity Tolerance in Plants. Int. J. Mol. Sci..

[55] Ltaeif H., Sakhraoui A., González-Orenga S., Faz A., Boscaiu M., Vicente Ó., Rouz S. (2021). Responses to Salinity in Four *Plantago* Species from Tunisia. Plants.

[56] Azizi M., Faz A., Zornoza R., Martinez-Martinez S., Acosta J.A. (2023). Phytoremediation Potential of Native Plant Species in Mine Soils Polluted by Metal(loid)s and Rare Earth Elements. Plants.

[57] Ioniță M.F., Dunca E.C., Radu S.M. (2026). Molecular and Cellular Mechanisms of Plant Responses to Heavy Metal Stress in Mining-Impacted Environments. Plants.

[58] Kalaji H.M., Jajoo A., Oukarroum A. (2016). Chlorophyll a fluorescence as a tool to monitor physiological status of plants under abiotic stress conditions. Acta Physiol. Plant..

[59] Sasi S., Venkatesh J., Daneshi R., Gururani M. (2018). Photosystem II Extrinsic Proteins and Their Putative Role in Abiotic Stress Tolerance in Higher Plants. Plants.

[60] Netondo G.W., Onyango J.C., Beck E. (2004). Sorghum and salinity: II. Gas exchange and chlorophyll fluorescence of sorghum under salt stress. Crop Sci..

[61] Taïbi K., Taïbi F., Abderrahim L.A., Ennajah A., Belkhodja M., Mulet J.M. (2016). Effect of salt stress on growth, chlorophyll content, lipid peroxidation and antioxidant defence systems in *Phaseolus vulgaris* L.. S. Afr. J. Bot..

[62] Havaux M. (2014). Carotenoid oxidation products as stress signals in plants. Plant J..

[63] Mehta D., Gamit S., Dudhagara D., Parmar V., Patel A., Vyas S. (2024). Carbohydrate accumulation patterns in mangrove and halophytic plant species under seasonal variation. Sci. Rep..

[64] Rao M.J., Duan M., Ikram M., Zheng B. (2025). ROS Regulation and Antioxidant Responses in Plants Under Air Pollution: Molecular Signaling, Metabolic Adaptation, and Biotechnological Solutions. Antioxidants.

[65] Keunen E., Peshev D., Vangronsveld J., Van Den Ende W., Cuypers A. (2013). Plant sugars are crucial players in the oxidative challenge during abiotic stress: Extending the traditional concept. Plant Cell Environ..

[66] Lohani N., Singh M.B., Bhalla P.L. (2022). Biological Parts for Engineering Abiotic Stress Tolerance in Plants. Biodes. Res..

[67] d’Alessandro S., Havaux M. (2019). Sensing β-carotene oxidation in photosystem II to master plant stress tolerance. New Phytol..

[68] Ibrahim M.H., Jaafar H.Z. (2012). Primary, secondary metabolites, H_2_O_2_, malondialdehyde and photosynthetic responses of *Orthosiphon stimaneus* Benth. to different irradiance levels. Molecules.

[69] Mishra N., Jiang C., Chen L., Paul A., Chatterjee A., Shen G. (2023). Achieving abiotic stress tolerance in plants through antioxidative defense mechanisms. Front. Plant Sci..

[70] Hussain T., Li J., Feng X., Asrar H., Gul B., Liu X. (2021). Salinity induced alterations in photosynthetic and oxidative regulation are ameliorated as a function of salt secretion. J. Plant Res..

[71] Ievina S., Karlsons A., Osvalde A., Andersone-Ozola U., Ievins G. (2023). Coastal Wetland Species Rumex hydrolapathum: Tolerance against Flooding, Salinity, and Heavy Metals for Its Potential Use in Phytoremediation and Environmental Restoration Technologies. Life.

[72] Wu B., Peng H., Sheng M., Luo H., Wang X., Zhang R., Xu F., Xu H. (2021). Evaluation of phytoremediation potential of native dominant plants and spatial distribution of heavy metals in abandoned mining area in Southwest China. Ecotoxicol. Environ. Saf..

[73] Zandi P., Schnug E. (2022). Reactive Oxygen Species, Antioxidant Responses and Implications from a Microbial Modulation Perspective. Biology.

[74] Mujeeb A., Abideen Z., Aziz I., Sharif N., Hussain M.I., Qureshi A.S., Yang H.H. (2023). Phytoremediation of Potentially Toxic Elements from Contaminated Saline Soils Using *Salvadora persica* L.: Seasonal Evaluation. Plants.

[75] Pirasteh-Anosheh H., Samadi M., Kazemeini S.A., Ozturk M., Ludwiczak A., Piernik A. (2023). ROS Homeostasis and Antioxidants in the Halophytic Plants and Seeds. Plants.

[76] Liu F., Xi M., Liu T., Wu X., Ju L., Wang D. (2024). The central role of transcription factors in bridging biotic and abiotic stress responses for plants’ resilience. New Crops.

[77] Kumar K., Debnath P., Singh S., Kumar N. (2023). An Overview of Plant Phenolics and Their Involvement in Abiotic Stress Tolerance. Stresses.

[78] Rao M., Zheng B. (2025). The Role of Polyphenols in Abiotic Stress Tolerance and Their Antioxidant Properties to Scavenge Reactive Oxygen Species and Free Radicals. Antioxidants.

[79] Gözcü S., Atmaca M. (2025). Antioxidant Potential and Phenolic Content of Plantago major. Curr. Health Sci. J..

[80] Souza M., Silva B., Badiale-Furlong E., Costa C. (2021). Phenolic Acid Profile, Quercetin Content, and Antioxidant Activity of Six Brazilian Halophytes. Handbook of Halophytes.

[81] Brzović P., Radman S., Mekinić I.G. (2025). Phenolic Antioxidants in the Adriatic Halophyte *Limbardacrithmoides*: Variation Across Phenological Stages. Foods.

[82] Jańczak-Pieniążek M., Cichoński J., Michalik P., Chrzanowski G. (2022). Effect of Heavy Metal Stress on Phenolic Compounds Accumulation in Winter Wheat Plants. Molecules.

[83] Jarin A.S., Khan M.A.R., Apon T.A., Islam M.A., Rahat A., Akter M., Anik T.R., Nguyen H.M., Nguyen T.T., Ha C.V. (2025). Plant Responses to Heavy Metal Stresses: Mechanisms, Defense Strategies, and Nanoparticle-Assisted Remediation. Plants.

[84] Ksouri R., Megdiche W., Debez A., Falleh H., Grignon C., Abdelly C. (2007). Salinity effects on polyphenol content and antioxidant activities in leaves of the halophyte *Cakile maritima*. Plant Physiol. Biochem..

[85] Chouari W. (2015). Land use and recent morphodynamics in the Ariana River sebkha watershed (North-eastern Tunisia). Confins.

[86] Chouari W. (2009). Environment and Natural Risks in Greater Tunis, Cartographic Approach. Ph.D. Thesis.

[87] Ghouma A., Aydi A., Rodríguez-Martín J.A., Gasmi M. (2022). Health risk assessment associated to heavy metal pollution levels in Mediterranean environment soils: A case study in the watershed of Sebkhet Ariana, Tunisia. Arab. J. Geosci..

[88] Ellouzi H., Oueslati S., Hessini K., Rabhi M., Abdelly C. (2021). Seed-priming with H_2_O_2_ alleviates subsequent salt stress by preventing ROS production and amplifying antioxidant defense in cauliflower seeds and seedlings. Sci. Hortic..

[89] Aydi S., Aydi S.S., Ben Salah G., Dekhil A., Rahmani R., Bouajila A., Mohamed N.A., Abdelly C. (2023). Phytoremediation potential of native plants: Biomonitoring approach in contaminated soils approach in contaminated soils. Not. Bot. Horti Agrobot..

[90] Venkateswaran P., Vellaichamy S., Palanivelu K. (2007). Speciation of heavy metals in electroplating industry sludge and wastewater residue using inductively coupled plasma. Int. J. Environ. Sci. Technol..

[91] Saha P., Shinde O., Sarkar S. (2017). Phytoremediation of industrial mines wastewater using water hyacinth. Int. J. Phytoremediat..

[92] Lichtenthaler H.K. (1987). Chlorophylls and carotenoids: Pigments of photosynthetic biomembranes. Methods Enzymol..

[93] Hodges D.M., DeLong J.M., Forney C.F., Prange R.K. (1999). Improving the thiobarbituric acid-reactive-substances assay for estimating lipid peroxidation in plant tissues containing anthocyanin and other interfering compounds. Planta.

[94] Sergiev I., Alexieva V., Karanov E. (1997). Effect of spermine, atrazine and combination between them on some endogenous protective systems and stress markers in plants. Compt. Rend. Acad. Bulg. Sci..

[95] Hatano T., Kagawa H., Yasuhara T., Okuda T. (1988). Two new flavonoids and other constituents in licorice root: Their relative astringency and radical scavenging effects. Chem. Pharm. Bull..

[96] Bradford M. (1976). A rapid and sensitive method for the quantitation of microgram quantities of protein utilizing the principle of protein-dye binding. Anal. Biochem..

[97] Oyaizu M. (1986). Studies on products of browning reaction. Jpn. J. Soc. Nutr. Diet..

[98] Ellouzi H., Sghayar S., Abdelly C. (2017). H_2_O_2_ seed priming improves tolerance to salinity, drought and their combined effect more than mannitol in *Cakile maritima* when compared to *Eutrema salsugineum*. J. Plant Physiol..

[99] Oktay M., Gülçin İ., Küfrevioğlu Ö.İ. (2003). Determination of in vitro antioxidant activity of fennel (*Foeniculum vulgare*) seed extracts. LWT Food Sci. Technol..

[100] Dewanto V., Wu X., Adom K.K., Liu R.H. (2002). Thermal processing enhances the nutritional value of tomatoes by increasing total antioxidant activity. J. Agric. Food Chem..

[101] Djeridane A., Yousfi M., Nadjemi B., Boutassouna D., Stocker P., Vidal N. (2006). Antioxidant activity of some Algerian medicinal plants extracts containing phenolic compounds. Food Chem..

[102] Maksimović Z., Malencić D., Kovacević N. (2005). Polyphenol contents and antioxidant activity of *Maydis stigma* extracts. Bioresour. Technol..

